# The epithelioid blue nevus: A rare intraoral nevomelanocytic tumor

**DOI:** 10.4103/0973-029X.80018

**Published:** 2011

**Authors:** Atef Hanna, Swati Y Rawal, Kenneth M Anderson, Yeshwant B Rawal

**Affiliations:** *Department of Pathology, College of Medicine, University of Tennessee Health Science Center, Memphis, TN*; 1 *Department of Periodontology, College of Dentistry, University of Tennessee Health Science Center, Memphis, TN,*; 2 *Department of Oral and Maxillofacial Pathology, College of Dentistry, University of Tennessee Health Science Center, Memphis, TN*

**Keywords:** Blue, epithelioid, melanocytic, mucosal, nevus

## Abstract

The epithelioid blue nevus (EBN) is considered a cutaneous marker of the Carney complex. Sporadic EBN has been reported in patients not exhibiting the Carney complex. The EBN does not exhibit unique clinical features that help to differentiate it from other lesions and is often provisionally diagnosed as an acquired melanocytic nevus, or a malignant melanoma. A 52-year-old African-American female had a 3-4 mm bluish macule of the left anterior hard palate. An excisional biopsy was performed to rule out an incipient melanoma. Formalin-fixed, hematoxylin and eosin-stained sections were examined microscopically. On the basis of histopathological features, a diagnosis of EBN was rendered. We document a case of the rare EBN affecting the oral mucosa. The patient did not exhibit any features associated with the Carney complex. Two years post-operatively, there is no evidence of a recurrent tumor.

## INTRODUCTION

The blue nevus is a distinct benign acquired tumor derived from dermal melanocytes.[1] It typically presents as an asymptomatic, blue-gray, smooth-surfaced macule or papule. While blue nevi occur chiefly on the skin, they may occasionally be seen in other sites, such as the nose,[2] genital tract[3] and oral cavity.[4,5]

Within the oral cavity, the blue nevus is the second most common type of melanocytic nevus, accounting for approximately 16% of all oral nevi. Blue nevi of the oral cavity may be seen anywhere from the second to the eighth decade of life with a mean age at diagnosis of 43 years. They occur most commonly on the hard palate and are diagnosed more frequently in males than females by a ratio of 2:1.[6]

Histologically, the blue nevus is classified as either common or cellular blue nevus.[1,7] In 1996, Carney and Ferreiro[8] described the epithelioid blue nevus (EBN) in patients with Carney complex, an autosomal dominant syndrome characterized by macular skin pigmentation, cardiac, cutaneous and soft tissue myxomas, endocrine overactivity and psammomatoid melanotic schwannomas. Sporadic cases of the EBN have been reported in patients not exhibiting the Carney complex[3,9] and, to our knowledge, only one case has been reported to date in the oral cavity.[10] We document a case of the rare EBN affecting the mucosa of the hard palate. The patient did not exhibit any features associated with the Carney complex.

## CASE REPORT

A 52-year-old African-American female presented for a routine dental examination. During the examination, a 3-4 mm bluish macule of the left anterior hard palate was noted. The patient’s medical history was significant for insulin-dependent diabetes mellitus. The diabetes was adequately controlled and she was otherwise in good health. The presence of the palatal lesion had been unknown to the patient. A working diagnosis of melanotic macule was given. An excisional biopsy was performed to rule out incipient melanoma. Routinely processed, formalin-fixed, hematoxylin and eosin-stained sections were examined.

**Figure 1:**
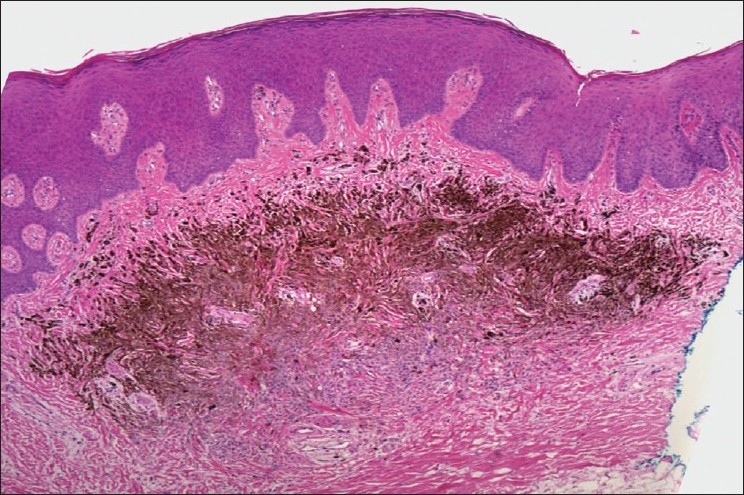
Dome-shaped tumor mass of the connective tissue separated from the surface epithelium by a band of collagen. The pigmented tumor is poorly circumscribed. H&E stain, 40× magnification

Microscopic examination showed a proliferation of pigmented tumor cells within the submucosa. A relatively clear grenz zone of connective tissue separated the tumor from the overlying epithelium. The tumor was neither encapsulated nor circumscribed. However, it did not demonstrate an infiltrating or penetrating periphery. Pigment production was most prominent among the superficial tumor cells, with those in the deeper tissues showing reduced intracytoplasmic pigmentation [Figure 1].

**Figure 2:**
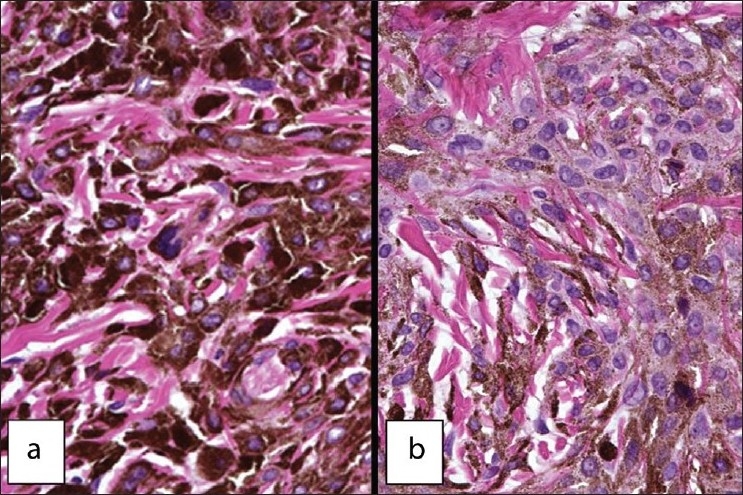
a) Coarse melanin granules obscure the epithelioid cell membranes. Intermingling of dense collagen bundles produces a checker board appearance. b) Epithelioid cells shown little pigmentation around the periphery of the cytoplasm. They also show a prominent nucleus and occasional dot-like nucleoli. The cell membranes are indistinct. H&E stain, 200× magnification

Upon closer examination, the epithelium did not demonstrate any nevomelanocytic activity among the basal cell layer or above. The heavily pigmented tumor cells were arranged in strands and sheets interspersed by thick bands of collagen. While the pigment-producing large cells were largely obscured by the intracytoplasmic melanin, their nuclei remained fairly prominent[Figure 2a]. Cells in the deeper portion of the tumor showed a reduction in the amount of melanin produced. This reduced tinctorial intensity revealed the epithelioid morphology of the cells with abundant amphophilic cytoplasm. The cytoplasmic membranes were indistinct. The nuclei showed intranuclear vacuolation with occasional pinpoint nucleoli[Figure 2b]. Mitotic activity and pleomorphism of the cells was not seen. No clusters or a “theque-like” arrangement of tumor cells was noted. On the basis of these histopathological features, a diagnosis of EBN was rendered. As the lesion was excised, no further intervention was necessary. Further, the patient did not exhibit features associated with the Carney complex. Almost 2 years post-operatively, there is no evidence of a recurrent tumor.

## DISCUSSION

The EBN is considered a cutaneous marker of the Carney complex. However, since the original description,[8] patients have been identified with EBN and no evidence of the Carney complex.[9] In these cases, the diagnosis of an EBN was established only upon microscopic examination of the biopsied tissue.

EBN do not exhibit any unique clinical features. Lesions are reported as darkly pigmented, dome-shaped nodules that are usually located on the skin of the extremities or trunk.[9] They are provisionally diagnosed as acquired melanocytic nevi, pigmented seborrheic keratosis, pigmented dermatofibroma or malignant melanoma.[10]

The only other case of an intraoral EBN was described as a dark blue, firm, sessile mass of the left buccal mucosa measuring 4x3 mm.[10] The tumor in our patient measured approximately 3 mm in diameter. It presented as a bluish macule of the left anterior hard palate and was provisionally diagnosed as a melanotic macule. Apparently, intraoral EBN may present as a nodule or a macule with bluish coloration. The clinical differential diagnosis therefore includes acquired melanocytic nevi, the common and cellular blue nevi, Spitz nevi, melanotic macule, melanoma, amalgam and graphite tattoo, pyogenic granuloma, vascular anomalies and varices and the peripheral giant cell granuloma. An excisional biopsy followed by histopathological examination of the tissue is required to arrive at a final diagnosis.

Histopathologically, the EBN requires differentiation from other tumors like the benign melanocytic nevi, the common and cellular blue nevi, the Spitz nevus and malignant melanoma.

Acquired melanocytic nevi evolve from junctional nevi to compound nevi and finally, to intramucosal nevi. In junctional nevi, clusters of nevus cells are arranged among the basal cells of the epithelium. As the nevus cells proliferate, clusters of these cells drop from the basal layer of the epithelium to nests of pigmented cells both within the basal layer of the epithelium and the superficial connective tissue, resulting in a compound nevus. Finally, in the intramucosal nevi, nevus cells are entirely within the connective tissue. The nevus cells in the superficial portions of the lesion are epithelioid and pigmented. They are arranged in clusters known as ‘theques’. The nevus cells of the middle portion of the tumor show scant pigment formation and resemble lymphocytes. Deeper nevus cells lack pigment and appear elongated and spindle shaped, resembling fibroblasts.[11]

Oral blue nevi are almost always seen on the palate. Histopathologically, the common blue nevus demonstrates collections of dendritic, elongated, slender melanocytes associated with a variably fibrotic stroma. The lesional cells form slender elongated processes and have variable amounts of delicate fine pigmentation within their cytoplasm.[12] The cellular blue nevus demonstrates a zoned appearance with cellular areas interspersed with areas resembling the common blue nevus. Plump melanocytes within the cellular areas exhibit rounded to oval nuclei in an eosinophilic cytoplasm. Pigment in the cellular areas is absent or moderate in intensity.[12]

The Spitz nevus typically develops during childhood. Microscopically, this lesion shows epithelioid cells that may appear bizarre and show increased mitotic activity. These cells are intermingled with spindle-shaped cells amidst ectatic superficial blood vessels. The transition of these cells from the basal layer to the connective tissue results in a compound nevus like architecture.[11]

Unlike these aforementioned nevi, the EBN demonstrates no junctional activity. The nevus cells are uniformly epithelioid throughout the lesion and there is no significant mitotic activity. Also, the cells do not tend to cluster in theques. Individual or groups of cells may be separated by a collagenous stroma but zonation, as seen in the cellular blue nevus, is not seen. Unlike the previous description of the intraoral EBN,[10] our case exhibited a reduction in the amount of pigment produced in the deeper portions of the tumor.

The EBN lacks junctional or pagetoid activity, cellular pleomorphism, or significant mitotic activity. Although it is unencapsulated and not circumscribed, it is symmetrical in appearance. These features help differentiate the EBN from a potential melanoma.

As EBN seldom measure more than a few millimeters in size, an excisional biopsy is curative. Further, a careful assessment of the patient should be carried out to exclude the possibility of the Carney complex.

We document a case of an EBN of the oral mucosa in a 52-year-old patient with no evidence of the Carney complex. Following an excisional biopsy, there is no evidence of recurrence after 2 years. The diagnosis was established only after histopathological examination of the biopsied tumor tissue.
